# 

**Published:** 1897-10-30

**Authors:** 


### EARLY EDITION.

**Notices of Births, Deaths, and Marriages** are inserted in this column at a charge of 2s. 6d. for an announcement not exceeding four lines.

DEATH.

On the 20th inst., suddenly, from hæmorrhage following phthisis of several years' duration, Georgina, the dearly loved eldest daughter of George Thomas and Rebecca Ridley, of 60, Pretoria Avenue, Walthamstow. (888)

### NOTICE TO CORRESPONDENTS.

All MS., Letters, Books for Review, and other matters intended for the Editor should be addressed The Editor, "The Hospital," The Hospital Building, 28 & 29, Southampton Street, Strand, W.C.

The Editor cannot undertake to return rejected MS., even when accompanied by stamped directed envelope.

**Notice to Advertisers and Subscribers.**

All Advertisements, Orders for Copies of The Hospital, and Business Communications should be addressed to **THE MANAGER, (Not to The Editor),** The Hospital, The Hospital Building, 28 & 29, Southampton Street, Strand, London, W.C.

The Cost of Subscription, post free, is as follows:—Per week, 2½d.; per quarter, 2s. 8d.; per half-year, 5s. 3d.; per annum, 10s. 6d. Foreign:—Per annum, 15s. 2d.; payable, in all cases, in advance.

**NOTICE.—Vol. XXII. of "The Hospital" (April, 1897, to September, 1897), in handsome cloth binding, is now on sale at the Office of this Journal, price 7s. 6d. Cases for binding the Numbers contained in this Volume, 1s. 6d. Index to Vol. XXII., 2½d., post free.**

**The Monthly Part for November will be ready on November 1st, Price 6d., by post 8d.**

### BOOKS RECEIVED.

The T. A. Davis Company.

"An Epitome of the History of Medicine." By Roswell Park, A.M., M.D.

William Reeves.

"Humanitarian Essays." Edited by Henry S. Salt.

Bailliere, Tindall, and Cox.

"Heart Disease." By Sir William H. Broadbent, Bart., M.D., F.R.S., and John F. Broadbent, M.A., M.D.

Isbister and Co.

"The Return to the Cross." By the Rev. W. Robertson Nicoll, M.A., LL.D.

**Periodicals and Pamphlets Received.**—Industries and Iron Woman's Signal, The Sun, Monthly Homœopathic Review, New Age Electrical Review, Hospital Saturday Fund Journal, Medical Press, Literary Digest, London, Sunday at Home, Boy's Own Paper, Local Government Journal, Sunday Hours, English Illustrated Magazine, Charity Organisation Review.

### Scraps and Gleanings.

So far the Southampton Jubilee Fund has amounted to £5,584.

About £1,500 has been subscribed towards the erection of a cottage hospital at Penrith.

The new medical block in connection with the Aberdeen Royal Infirmary has nearly been completed.

It is proposed to open a convalescent home for children in connection with the Charnwood Forest Convalescent Home.

Sixty patients were admitted to the Fleetwood Cottage Hospital in 1896-7. The income for the year amounted to £304.

A sum of £300 has been bequeathed towards the cost of a cottage hospital at Carshalton, provided the building be erected within twelve months.

The amount of the Newbury Hospital Saturday Collection this year was £131, as compared with £145 in 1896, £129 in 1895, £137 in 1894, and £132 in 1893.

The building of the new infectious hospital for Worcester is progressing rapidly. It will contain accommodation for about 40 patients, and will cost over £10,000.

Mr. Passmore Edwards has generously offered to erect a new home for the Friendly Societies' Convalescent Home at Herne Bay at a cost of not less than £6,000.

The number of patients treated at the Royal Infirmary, Derby, showed a large increase in 1896-7 as compared with 1895-6. The in-patients last year numbered 1,682, and in the previous year 1,418; out patients 6,773, as against 6,333; and casualties 4,304, as against 3,739. The daily average number of beds occupied increased from 115 to 136.

From the balance-sheet of the building fund of the Fleetwood Cottage Hospital, it appears that the cost of the building was £2,493, or with additional furnishing £3,602.

The total receipts at the Stratford-on-Avon Hospital (including a balance of £13 brought forward) amounted to £1,068, and the expenditure to £1,134, leaving a deficit of £66.

Quite recently a subscriber to a hospital at the annual meeting suggested that possibly the matron did not attend to her duties very well, as she had been seen about the streets on a bicycle! Need we add that this happened in Scotland.

Including 67 accident cases, 255 in-patients were treated at the Lowestoft Hospital in 1896-7. One thousand, two hundred and fifty-five out-patients, 375 casualties, and 525 dental cases were also under treatment. The income amounted to £1,418, and the expenditure to £1,391.

The report by Miss Sharman on the Orphan's Home, Austral Street, S.E., says that the principal feature of interest in regard to the home during the past year has been the opening of the Convalescent branch at Eastbourne and the Educational branch at Stockwell. This brings the number of homes up to seven. The report also states that the rules under which the homes have been worked are four, "No voting, no begging, no debt, and no managing expenses."

The managers of the Royal Infirmary at Edinburgh appealed recently against the assessment of the building for local purposes. The buildings were assessed at £6,180, and it was claimed that the hospital should only be assessed at a nominal sum. After hearing evidence, the Bench fixed the valuation at £5,000. The Royal Hospital for Sick Children, which appealed at the same time against an assessment of £1,500, was re-valued at £1,200.

### The Student of Medicine.

### APPOINTMENTS.

C. Berkeley, M.B., B.C.Cantab., M.R.C.P., M.R.C.S., appointed Out patient Physician to the Chelsea Hospital for Women. Dr. T. Dixon, appointed Medical Officer of Health to the Earsdon Urban District Council. H. B. Gardiner, M.R.C.S.-Eng., L.R.C.P.Lond., appointed Assistant Anæsthetist to Charing Cross Hospital, and Anæsthetist to the Male Lock Hospital. W. R. Hamilton, L.R.C.P., L.R.C.S.I., appointed Medical Officer to the Workhouse of the Edenderry Union. G. C. Hancock, L.R.C.P.Lond., M.R.C.S., appointed Assistant Medical Officer to the Port of London Sanitary Authority. Dr. F. Harris, appointed Medical Officer of Health for the Borough of St. Helens. G. Hope, L.R.C.P., M.R.C.S., L.S.A., D.P.H., appointed Medical Officer of Health for the Hanwell Urban District Council. G. A. Kenyon, M.B.Lond., L.R.C.P., M.R.C.S., appointed Medical Officer of Health to the Malpas Rural District. A. V. Leche, L.R.C.P.Edin., M.R.C.S.Eng., appointed Medical Officer of Health to the Axbridge Rural District Council. F. Martin, M.R.C.S., L.R.C.P., appointed Medical Officer of Health for the Borough of Brighouse. C. E. Paget, M.R.C.S.Eng, L.R.C.P.Lond., D.P.H.Eng., appointed County Medical Officer of Health for Northampton. E. F. Potter, M.D.Brux., M.R.C.S., L.R.C.P., appointed Assistant Surgeon to the London Throat Hospital. H. Stott, L.R.C.P.Lond., M.R.C.S.Eng., appointed Medical Officer of Health to the Seaford Urban District Council. J. A. Swindale, M.B., B.S.Durh., appointed Assistant House Surgeon to the Guest Hospital, Dudley, Worcestershire. E. Waggett, M.B, appointed Assistant Surgeon to the London Throat Hospital. G. W. Wigin, M.R.C.S.Eng., L.S.A., appointed Medical Officer of Health to the Methley Urban District Council.

